# Impact of a multi-step testing algorithm on hospital-onset *Clostridioides difficile* rates and clinical outcomes

**DOI:** 10.1017/ash.2025.180

**Published:** 2025-05-19

**Authors:** Matthew A. Moffa, Dustin R. Carr, Nathan R. Shively, Adriana Betancourth, Nitin Bhanot, Zaw Min, Charmaine Abalos, Arshpal Gill, Salman Bangash, Thomas L. Walsh

**Affiliations:** 1 Medicine Institute and Division of Infectious Diseases, Allegheny Health Network, Pittsburgh, PA, USA; 2 Department of Pharmacy, Allegheny Health Network, Pittsburgh, PA, USA

## Abstract

**Objective:**

To evaluate the impact of implementing a multi-step *Clostridioides difficile* infection (CDI) testing algorithm on hospital-onset (HO)-CDI rates and clinical outcomes.

**Design:**

Retrospective pre-intervention/post-intervention study.

**Setting:**

Two academic hospitals in Pittsburgh, Pennsylvania.

**Methods:**

In the pre-intervention period, a standalone polymerase chain reaction (PCR) assay was used for diagnosing CDI. In the post-intervention period, positive PCR assays were reflexed to a glutamate dehydrogenase antigen test and an enzyme immunoassay for toxin A/B.

**Results:**

The implementation of a multi-step testing algorithm resulted in a significant reduction in HO-CDI cases per 10,000 patient days from 5.92 to 2.36 (*P* < 0.001). Despite the decrease in reportable HO-CDI cases, there were no significant differences in clinical outcomes such as hospital length of stay, intensive care unit admissions, and treatment courses. In addition, there was a significant reduction in all-cause 30-day readmissions in the post-intervention group, though CDI-related readmissions remained similar.

**Conclusions:**

The multi-step testing algorithm significantly reduced HO-CDI rates without compromising clinical outcomes. The study supports the use of a multi-step CDI testing algorithm to assist healthcare providers with CDI management decisions and potentially to reduce financial penalties burdened on healthcare systems.

## Introduction

*Clostridioides difficile* infection (CDI) represents a significant challenge in healthcare settings due to its morbidity, mortality, and associated healthcare costs. The Centers for Disease Control and Prevention has declared CDI to be an urgent threat and estimated 223,900 cases in hospitalized patients resulting in 12,800 deaths and 1 billion dollars in attributable healthcare costs in 2017.^1^ Due to this significant health and economic burden, the US Department of Health and Human Services established an action plan to reduce hospital-onset (HO) CDI by establishing a reportable HO-CDI quality metric linking financial incentives or penalties to hospitals’ performance.^2^ HO-CDI is defined based on the National Healthcare Safety Network (NHSN) Laboratory Identified event as a positive *C. difficile* test result performed on a stool specimen collected ≥4 days after inpatient admission to the facility. When a multi-step testing algorithm for CDI is performed on the same stool specimen, the finding of the last test performed on the specimen as shown on the final report in the patient’s medical record will determine if the CDI-positive laboratory assay definition is met.^3^ In October 2016, the quality metric went into effect and was associated with a 6% decline in HO-CDI rates in the immediate post-policy quarter and a 4% decline in slope per quarter.^4^

One of the complicating factors with the definition of HO-CDI is the lack of standard diagnostic testing among different healthcare systems. Diagnostic methods often struggle with balancing sensitivity and specificity, leading to potential overdiagnosis or underdiagnosis of CDI, which can affect patient outcomes and financial penalties. In the late 2000s, many laboratories switched from toxin tests to molecular nucleic acid amplification tests (NAAT), such as polymerase chain reaction (PCR), due to increased sensitivity of molecular tests.^5^ This change led to a >50% increase in reporting rates of healthcare-associated CDI.^6,7^ However, using standalone molecular tests may detect colonization rather than true infection, potentially leading to unnecessary treatment and increased healthcare costs.^8^ As a result, the Infectious Diseases Society of America clinical practice guidelines recommend the use of a stool toxin test as part of a multi-step algorithm rather than a molecular test alone when there are no pre-agreed institutional criteria for patient stool submission.^9^ Algorithms could include a glutamate dehydrogenase (GDH) antigen test plus toxin enzyme immunoassay (EIA) test, GDH antigen test plus toxin EIA test arbitrated by a NAAT test, or a NAAT test plus toxin EIA test. Based on the current NHSN definition, some centers have shown that using a multi-step algorithm with a PCR test followed by a less sensitive, but more specific, toxin EIA test can decrease the reportable rate of HO-CDI since the last test is what NHSN requires for its HO-CDI definition.^10–12^

Our healthcare system recently changed diagnostic strategies from a standalone PCR to a multi-step testing algorithm. This consists of an initial PCR; if the PCR test result is negative, no further laboratory testing is performed. If *C. difficile* PCR testing is positive, GDH antigen test and toxin A/B EIA testing are performed. The goal of this study was to evaluate the impact the multi-step algorithm had on HO-CDI rates and clinical outcomes.

## Methods

### Study design

We conducted a retrospective pre-intervention/post-intervention study comparing HO-CDI rates and clinical outcomes before and after implementation of a multi-step CDI testing algorithm. The algorithm went live on 07/25/2022. The pre-intervention period was 07/01/2021 to 07/24/2022 and the post-intervention period was 07/25/2022 to 03/31/2023.

### Study setting

This study was conducted at 2 academic hospitals within the Allegheny Health Network (AHN) located in Pittsburgh, Pennsylvania. Allegheny General Hospital (AGH) is a 631-bed quaternary care teaching hospital with approximately 22,000 inpatient admissions yearly. West Penn Hospital (WPH) is a 317-bed community-based teaching hospital with nearly 6800 inpatient admissions annually. Exempt status was granted by the AHN Institutional Review Board.

### Intervention

**Figure 1.**
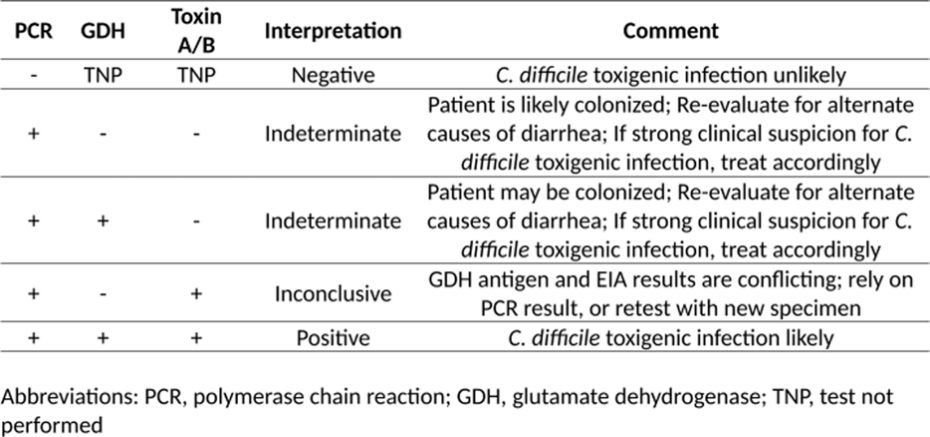
*C. difficile* test result interpretations reported in the electronic medical record.

In the pre-intervention period, the microbiology lab for both hospitals utilized the Cepheid Xpert® *C. difficil*e PCR assay (Cepheid Inc., Sunnyvale, CA) as a standalone test for suspected CDI cases. In the post-intervention period, a positive PCR assay was then reflexed to a GDH antigen test and toxin A/B EIA test utilizing the *C. DIFF QUIK CHEK COMPLETE*® (TECHLAB Inc., Blacksburg, VA). Results were reported in the electronic medical record (EMR) only after all tests were completed. These results included interpretive comments displayed in the EMR that were created in collaboration by the microbiology lab and division of Infectious Diseases (Figure 1). Decisions on treatment were made solely at the discretion of the treating medical providers. Once the intervention went live, a memo was distributed to all medical staff and education was provided by dissemination of an antimicrobial stewardship newsletter detailing the rationale of this change and testing interpretations. During both the pre- and post-intervention periods, stool for CDI testing was only processed for testing by the lab if it was a 6 or 7 on the Bristol stool scale. Also, during both time periods, CDI testing order entry in the EMR required the user to answer questions regarding stool frequency and bowel regimen. Answering that the patient had less than 3 loose bowel movements or that the patient was taking stool softeners or laxatives prompted the EMR to discourage, but not restrict, CDI test ordering. No other CDI interventions were initiated during this study and oral vancomycin was recommended as first-line therapy in our institutional CDI guidance during both time periods.

### Data collection

Patients ≥18 years of age were identified and included for analysis if they had a positive *C. difficile* PCR assay during an inpatient admission to either AGH or WPH during the defined study dates for either study period. Patients with multiple hospitalizations with a positive *C. difficile* PCR assay had each hospitalization included for analysis. If multiple PCR assays were positive during a single admission or within 14 days between a readmission, only the first encounter was recorded. Data were extracted from the EMR by the study investigators using a standard data collection tool. Demographic information, patient comorbidities, admission and discharge dates, laboratory and clinical data, inpatient and outpatient antimicrobial therapy, and subsequent inpatient admissions during the 30 days following hospital discharge were collected.

### Study outcomes and definitions

The primary outcome of this study was to evaluate the impact of a multi-step CDI testing algorithm on HO-CDI rates. Secondary outcomes were the clinical impact of the multi-step algorithm on patient care in the pre-intervention vs. post-intervention periods, as well as the clinical impact of a positive EIA toxin vs negative EIA toxin on patient care in the post-intervention period.

HO-CDI was defined using NHSN criteria.^3^ When using the multi-step algorithm in the post-intervention period, the finding of the last test performed (EIA toxin A/B) on the stool specimen determined if the CDI-positive laboratory assay definition was met. A treatment course for CDI was defined as receipt of at least 5 days of antibiotics targeting CDI.

### Statistical analysis

The normality of continuous variables was assessed using the Kolmogorov-Smirnov test. The two-sample *t*-test or Wilcoxon rank-sum test were used to assess continuous variables depending on distribution. Categorical variables were assessed using the *χ* ^2^ test or Fisher’s exact test as appropriate. *P* < 0.05 was considered statistically significant. Statistical analysis was performed using R, version 4.2.1 (R Foundation for Statistical Computing, Vienna, Austria).

## Results

**Figure 2.**
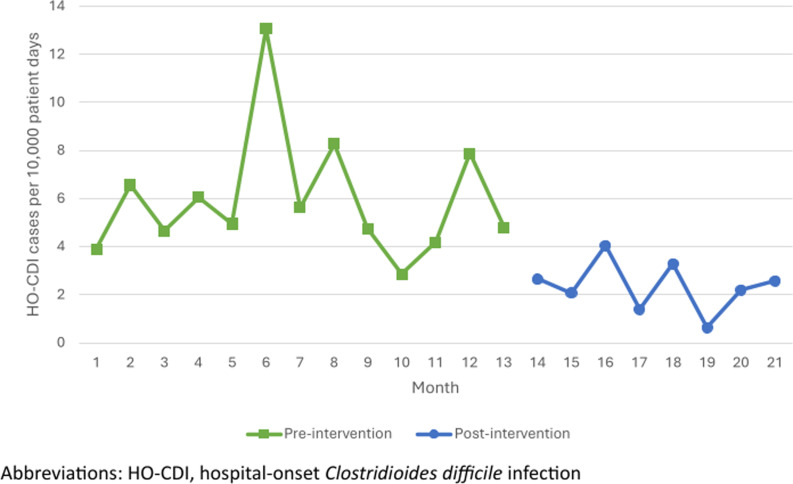
HO-CDI rate by month.

A total of 244 patient admissions with a positive *C. difficile* PCR assay were included in the pre-intervention period and 150 patient admissions with a positive *C. difficile* PCR assay were included in the post-intervention period (including both community-onset and HO-CDI during each time period). The rate of HO-CDI cases per 10,000 patient days decreased significantly after the implementation of the multi-step algorithm (5.92 vs 2.36, *P* < 0.001, Figure 2). Of those 150 patient encounters in the post-intervention period, 58 had a positive PCR after day 4 of admission, which prior to implementation of the multi-step algorithm would have resulted in 58 cases of HO-CDI meeting the NSHN definition. However, only 27 of those 58 cases had a positive EIA toxin, resulting in a 54% reduction in reportable HO-CDI cases to NHSN in the post-intervention period (*P* < 0.001).

Baseline comparisons between the pre- and post-intervention groups are found in Table 1. There was no significant difference in age, sex, race, or comorbidities. Clinical comparisons between the two groups are found in Table 2. There was no significant difference in hospital length of stay (LOS), intensive care unit (ICU) admissions and LOS, maximum white blood cell (WBC) count, abdominal pain, or bowel movement frequency. No significant difference was found between the two groups regarding CDI treatment courses. Repeat CDI testing (both inpatient and outpatient) and subsequent treatment in the next 90 days was also similar between the two groups. There was a significant reduction in all cause 30-day readmissions in the post-intervention group, though readmissions related to CDI was similar between the two groups. Regarding subspecialty consultations, significantly more patients had an infectious disease (ID) consult in the post-intervention period. ID and Gastroenterology (GI) consultants were more likely to deem a positive PCR test as colonization in the post-intervention period, though a similar rate of patients deemed to be colonized were treated in the two study periods.

**Table 1.** Baseline demographics of patients admitted with a positive *C. difficile* PCR assay

	Pre-intervention N = 244	Post-intervention N = 150	*P* value
Age, years, mean ± SD	62.6 ± 14.9	63.9 ± 15.8	0.443
Sex, female	138 (56.6)	80 (53.3)	0.532
**Race**			0.785
White	211 (86.5)	131 (87.3)	
Black	31 (12.7)	17 (11.3)	
Other/Unknown	2 (0.8)	2 (1.3)	
Charlson Comorbidity Index, mean ± SD	5.7 ± 3.3	5.6 ± 3.2	0.774

Note: All data are reported as n (%) unless specified otherwise.

Abbreviations: (PCR), polymerase chain reaction; (SD), standard deviation.

**Table 2.** Clinical findings of patient admissions with a positive *C. difficile* PCR assay during the pre- and post-intervention time periods

	Pre-intervention N = 244	Post-intervention N = 150	*P* value
Hospital LOS, days, mean ± SD	13.9 ± 15.9	13 ± 14.1	0.556
Time from admission to positive PCR, days, mean ± SD	6.6 ± 9.9	5.2 ± 9	0.152
Positive PCR ≥4 days after admission	112 (45.9)	58 (38.7)	0.159
Clinical findings during the 2 days before and after positive PCR			
Highest WBC, k/mcL, mean ± SD	15.6 ± 10.2	14.9 ± 11.1	0.491
Abdominal pain	104 (42.6)	68 (45.3)	0.598
Abdominal distention	42 (17.2)	27 (18)	0.842
*Bowel movements per day*			0.728
Less than 3	40 (16.4)	24 (16)	
3 or more	177 (72.5)	113 (75.3)	
Not documented	27 (11.1)	13 (8.7)	
Received 5 days or more of CDI treatment inpatient or at discharge	213 (87.3)	124 (82.7)	0.205
Spent time in ICU	76 (31.1)	58 (38.7)	0.126
ICU LOS, days, mean ± SD	15.9 ± 15.2	13 ± 13.6	0.251
ID consulted	77 (31.6)	63 (42)	**0.035**
If yes, evaluated as colonization	11 (14.3)	20 (31.7)	**0.011**
Evaluated as colonization but treated, n/N (%)	5/11 (45.5)	9/20 (45)	1
GI consulted	48 (19.7)	42 (28)	0.056
If yes, evaluated as colonization	1 (2.1)	7 (16.7)	**0.023**
Evaluated as colonization but treated, n/N (%)	0/1 (0)	2/7 (28.6)	1
Testing repeated within 90 days	44 (18)	24 (16)	0.604
If yes, days from initial PCR, mean ± SD	39.4 ± 22.1	42.6 ± 23.9	0.583
If yes, PCR positive result	29 (65.9)	18 (75)	0.438
If yes, EIA toxin result			**<0.001**
Negative	4 (9.1)	11 (45.8)	
Positive	0 (0)	9 (37.5)	
Not performed	40 (90.9)	4 (16.7)	
Subsequent treatment in next 90 days for CDI	26 (10.7)	17 (11.3)	0.834
30 day readmission	64 (26.2)	26 (17.3)	**0.041**
Time to readmission, days, mean ± SD	14.2 ± 9.4	12.9 ± 7.2	0.506
Related to CDI	14 (21.9)	4 (15.4)	0.572

Note: All data are reported as n (%) unless specified otherwise.

Abbreviations: LOS, length of stay; PCR, polymerase chain reaction; SD, standard deviation; WBC, white blood cells; ICU, intensive care unit; ID, infectious diseases; GI, gastroenterology; EIA, enzyme immunoassay.

In the post-intervention group, 134 patients (89.3%) had a positive EIA GDH while only 52 patients (34.7%) had a positive EIA toxin (Table 3). Patients with a positive EIA toxin had significantly longer hospital LOS, more ICU admissions, higher peak WBC counts, and more frequent bowel movements. Every toxin-positive patient was treated for CDI, whereas 73.5% of toxin-negative patients were treated. ID consultants were significantly more likely to label toxin-negative patients as colonization compared to toxin-positive patients (51.4% vs 3.8%, *P* < 0.001). In the post-intervention period, ID consultants were also significantly more likely to label toxin-negative patients as colonization than GI consultants (51.4% vs 23.3%, *P* = 0.025). Repeat testing within 90 days was performed similarly in both groups. Of the 16 toxin-negative patients that had repeat testing, 10 remained PCR positive and 4 of those 10 became toxin positive. Of the 8 toxin-positive patients that had repeat testing, all remained PCR positive, and 3 reverted to toxin-negative. Retreatment and readmission rates did not differ between the toxin-negative and toxin-positive groups. Among the 98 patients that were toxin negative, there was no significant difference in 30-day readmission rates among those that were treated for CDI vs those not treated ((13/72 (18.1%) vs 2/26 (7.7%), *P* = 0.341)). There was also no significant difference in subsequent treatment within 90 days among those who were treated for CDI initially vs those not treated initially ((6/27 (8.3%) vs 3/26 (11.5%), *P* = 0.696)).

**Table 3.** Clinical findings of patient admissions with a positive *c. difficile* pcr assay during the post-intervention time period based on enzyme immunoassay toxin result

	PCR+/EIA- N = 98	PCR+/EIA+ N = 52	*P* value
Hospital LOS, days, median (IQR)	6.6 (3, 15)	12.8 (5.2, 21.5)	**0.006**
Time from admission to positive PCR, days, median (IQR)	2 (1, 5)	4.5 (1, 10.3)	**0.008**
Positive PCR ≥ 4 days after admission	31 (31.6)	27 (51.9)	**0.015**
Clinical findings during the 2 days before and after positive PCR			
Highest WBC, k/mcL, median (IQR)	10.8 (6.8, 17.3)	14.3 (10.5, 20.4)	**0.047**
Abdominal pain	44 (44.9)	24 (46.2)	0.883
Abdominal distention	21 (21.4)	6 (11.5)	0.134
Bowel movements per day			**0.016**
Less than 3	19 (19.4)	5 (9.6)	
3 or more	67 (68.4)	46 (88.5)	
Not documented	12 (12.2)	1 (1.9)	
Received 5 days or more of CDI treatment inpatient or at discharge	72 (73.5)	52 (100)	**<0.001**
Spent time in ICU	30 (30.6)	28 (53.8)	**0.005**
ICU LOS, days, mean ± SD	11.2 ± 8.5	15 ± 17.4	0.299
ID consulted	37 (37.8)	26 (50)	0.148
If yes, evaluated as colonization	19 (51.4)	1 (3.8)	**<0.001**
Evaluated as colonization but treated, n/N (%)	8/19 (42.1)	1/1 (100)	0.45
GI consulted	30 (30.6)	12 (23.1)	0.328
If yes, evaluated as colonization	7 (23.3)	0 (0)	0.164
Evaluated as colonization but treated, n/N (%)	2/7 (28.6)	–	–
Testing repeated within 90 days	16 (16.3)	8 (15.4)	0.881
If yes, days from initial PCR, mean ± SD	45.7 ± 25.2	36.5 ± 21	0.359
If yes, PCR positive result	10 (62.5)	8 (100)	0.066
If PCR positive, EIA toxin positive result	4 (33.3)	5 (62.5)	0.637
Subsequent treatment in next 90 days for CDI	9 (9.2)	8 (15.4)	0.254
30 day readmission	15 (15.3)	11 (21.2)	0.368
Time to readmission, days, mean ± SD	13.7 ± 8	11.8 ± 6.3	0.5
Related to CDI	1 (6.7)	3 (27.3)	0.279

Note: All data are reported as n (%) unless specified otherwise.

Abbreviations: LOS, length of stay; IQR, interquartile range; PCR, polymerase chain reaction; SD, standard deviation; WBC, white blood cells; ICU, intensive care unit; ID, infectious diseases; GI, gastroenterology; EIA, enzyme immunoassay.

## Discussion

The implementation of a multi-step testing algorithm for CDI had a considerable impact on HO-CDI rates and patient management in our healthcare system. We observed a significant reduction in HO-CDI cases per 10,000 patient days from 5.92 to 2.36 (*P* < 0.001). This finding is consistent with previous studies that have shown that multi-step algorithms can lower CDI rates by improving diagnostic specificity and reducing the likelihood of detecting asymptomatic colonization rather than true infection.^10–12^ The reduction in HO-CDI rates can be attributed to the increased specificity of the multi-step algorithm, which incorporates both PCR and EIA toxin A/B. This approach mitigates the risk of overdiagnosis associated with standalone molecular tests that may detect colonization. The NHSN definition of HO-CDI, which relies on the final test result, underscores the importance of using a diagnostic strategy that balances sensitivity and specificity.^3^ In the post-intervention period, 54% of PCR-positive patients that previously would have met the NHSN definition of HO-CDI were negative for EIA toxin, thus reducing the number of reportable cases and potential financial penalties levied against our healthcare system. Our findings support current guidelines recommending multi-step algorithms to improve CDI diagnosis and reporting accuracy.^9^

Despite the significant reduction in reportable HO-CDI cases, our study found no significant differences in clinical outcomes between the pre- and post-intervention periods (Table 2). Key metrics such as hospital LOS, ICU admissions, maximum WBC count, abdominal pain, bowel movement frequency, repeat testing within 90 days, and subsequent treatment for CDI in the next 90 days remained comparable. There was a significantly lower all-cause 30-day readmission rate in the post-intervention group, though readmission related to CDI were similar. This suggests that the multi-step algorithm did not adversely affect patient care quality or outcomes, aligning with previous research indicating that more specific diagnostic criteria do not compromise clinical management.^12,13^ Interestingly, we found that clinicians continued to treat PCR-positive patients at the same rate between the pre- and post-intervention time periods (87% vs 83%, *P* = 0.205), which may be an important factor why clinical outcomes remained similar. This may have been due to a combination of unfamiliarity with a new test and the clinician fear of withholding an antibiotic, as well as the multi-step algorithm recommending treatment of EIA toxin-negative patients where strong clinical suspicion for CDI infection persisted. However, significantly less EIA toxin-negative patients in the post-intervention arm were treated compared to EIA toxin-positive patients (73.5% vs 100%, *P* < 0.001). Thus, though the treatment rate of PCR-positive patients stayed the same between the pre- and post-intervention time periods, utilizing the multi-step algorithm to identify toxin-negative patients in the post-intervention period helped providers identify which patients they could safely defer treatment.

There was an increase in ID and GI consultations in the post-intervention period, suggesting that the multi-step algorithm prompted more thorough clinical evaluations to assist with interpretation of indeterminate results. In the post-intervention period, both ID and GI consultants were more likely to document positive PCR results as indicative of colonization rather than infection (26 of the 27 cases documented as colonization had a negative (EIA) toxin). Despite consultants documenting more PCR-positive cases as colonization in the post-intervention period, the proportion of those cases considered colonized that received (CDI) treatment remained the same between the 2 groups (41.7% vs 40.7%, *P* = 1)

Our analysis of patients with positive PCR results in the post-intervention period revealed significant differences in management based on EIA toxin results (Table 3). Patients with positive EIA toxin tests were more likely to receive CDI treatment, reflecting the higher specificity and clinical application of toxin detection. ID consultants were more likely to label toxin-negative patients as colonized compared to toxin-positive patients (*P* < 0.001) and did so at a higher rate than GI consultants (*P* = 0.025). This stratification underscores the utility of the multi-step algorithm in refining CDI diagnosis and guiding appropriate treatment decisions. The subgroup analysis also revealed that repeat testing within 90 days was performed similarly regardless of initial toxin status, with some toxin-negative patients converting to toxin-positive on subsequent testing, and vice versa. This finding highlights the potential for intermittent toxin shedding and the need for ongoing clinical vigilance in managing patients with suspected CDI. Our study also found that the treatment of toxin-negative patients, compared to toxin-negative patients that did not receive CDI treatment, did not reduce the need for subsequent treatment in the next 90 days (8.3% vs 11.5%, *P* = 0.696), nor reduce 30-day readmission rates (18.1% vs 7.7%, *P* = 0.341). This suggests that withholding antibiotics in toxin-negative patients deemed colonized may not adversely affect outcomes. However, there may still be some PCR-positive/toxin-negative patients that may benefit from treatment.^14^ As such, it is always important to use any diagnostic strategy as just one part of a comprehensive approach to clinical decision-making.

Our study had some important limitations. The retrospective design may have introduced biases related to data collection and interpretation. The study was conducted over a relatively short period and involved a specific patient population from two academic hospitals in one geographic region. A larger sample size and longer study duration could provide more comprehensive insights into the long-term effects of the multi-step algorithm, as clinicians may be slow to adopt withholding treatment of select PCR-positive patients. The decision to treat patients was left to the discretion of the treating medical providers, which could introduce variability in treatment approaches. This variability might affect the consistency of clinical outcomes and the interpretation of the results. The study also highlighted the potential for intermittent toxin shedding, as some toxin-negative patients converted to toxin-positive on subsequent testing, and vice-versa. This complexity in toxin detection underscores the need for ongoing clinical vigilance but also introduces a layer of complexity in interpreting the results. Our study also did not assess for any mortality differences. Finally, our findings may not be relatable to centers that have implemented a multi-step algorithm utilizing different tests or order of tests, particularly those that do not include NAAT testing as the initial step. Addressing these limitations in future studies could enhance the understanding of the multi-step testing algorithm’s impact on CDI diagnosis and management.

The shift to a multi-step testing algorithm for CDI in our healthcare system led to a significant reduction in reportable HO-CDI cases without compromising clinical outcomes. Our findings reinforce the value of multi-step testing algorithms in managing CDI and highlight the importance of ongoing education and collaboration among healthcare providers to optimize patient care.
